# The function of BoTCP25 in the regulation of leaf development of Chinese kale

**DOI:** 10.3389/fpls.2023.1127197

**Published:** 2023-04-18

**Authors:** Jiajing Zeng, Mengyu Yang, Jing Deng, Dongyang Zheng, Zhongxiong Lai, Gefu Wang-Pruski, Xu XuHan, Rongfang Guo

**Affiliations:** ^1^ College of Horticulture, Fujian Agriculture and Forestry University, Fuzhou, China; ^2^ Department of Plant, Food, and Environmental Sciences, Faculty of Agriculture, Dalhousie University, Truro, NS, Canada; ^3^ Faculté des sciences et de la technologie, Institut de la Recherche Interdiciplinaire de Toulouse (IRIT-ARI), Toulouse, France

**Keywords:** *BoTCP25*, Chinese kale, leaf, *BoNGA3*, metamorphic leaf

## Abstract

XG Chinese kale (*Brassica oleracea* cv. ‘*XiangGu*’) is a variety of Chinese kale and has metamorphic leaves attached to the true leaves. Metamorphic leaves are secondary leaves emerging from the veins of true leaves. However, it remains unknown how the formation of metamorphic leaves is regulated and whether it differs from normal leaves. *BoTCP25* is differentially expressed in different parts of XG leaves and respond to auxin signals. To clarify the function of BoTCP25 in XG Chinese kale leaves, we overexpressed *BoTCP25* in XG and *Arabidopsis*, and interestingly, its overexpression caused Chinese kale leaves to curl and changed the location of metamorphic leaves, whereas heterologous expression of *BoTCP25* in *Arabidopsis* did not show metamorphic leaves, but only an increase in leaf number and leaf area. Further analysis of the expression of related genes in Chinese kale and *Arabidopsis* overexpressing *BoTCP25* revealed that BoTCP25 could directly bind the promoter of *BoNGA3*, a transcription factor related to leaf development, and induce a significant expression of *BoNGA3* in transgenic Chinese kale plants, whereas this induction of *NGA3* did not occur in transgenic *Arabidopsis*. This suggests that the regulation of Chinese kale metamorphic leaves by BoTCP25 is dependent on a regulatory pathway or elements specific to XG and that this regulatory element may be repressed or absent from *Arabidopsis*. In addition, the expression of miR319’s precursor, a negative regulator of *BoTCP25*, also differed in transgenic Chinese kale and *Arabidopsis*. miR319’s transcrips were significantly up-regulated in transgenic Chinese kale mature leaves, while in transgenic *Arabidopsis*, the expression of miR319 in mature leaves was kept low. In conclusion, the differential expression of *BoNGA3* and miR319 in the two species may be related to the exertion of BoTCP25 function, thus partially contributing to the differences in leaf phenotypes between overexpressed *BoTCP25* in *Arabidopsis* and Chinese kale.

## Introduction

Leaf development in plants is precisely regulated and the mechanisms of leaf ontogenesis and related genes have been successively identified (Du et al., 2018). Leaf blades vary drastically in different plant types. Depending on the number of leaflets attached to the petiole, they can be divided into simple and compound leaves, both of which have commonalities and differences in their regulatory mechanisms (Idan et al., 2010; Bar and Ori, 2014). At present, metamorphic leaves are found both on simple and compound leaves. The formation of metamorphic leaves of plants is closely related to their functions, for example, the variation of leaf shape in the genus *Balsamina* can affect the feeding selectivity of herbivorous insects (Higuchi and Kawakita, 2019). The formation of metamorphic leaves is similar to that of common leaves, which also develop from the leaf primordia (Wang and Chen, 2013). The difference is that the leaf primordia of metamorphic leaves often develop from a bunch of cells with allotropic properties and their positions are not fixed (Ori et al., 2007). However, little is known about the regulatory mechanisms of metaplastic leaf formation.

*TCP* (*TEOSINTE BRANCHED1* (Doebley et al., 1995), *CYCLOIDEA* (Da et al., 1996), *PROLIFERATING CELL FACTORS* (Cubas et al., 1999)) is a plant-specific class of transcription factors that regulate cell growth and proliferation (Martin-Trillo and Cubas, 2010). With the development of molecular biology techniques, the *TCP* gene family has been successively identified in species such as *Arabidopsis* (Yao X et al., 2007), tomato (Parapunova et al., 2014), potato (Xiao et al., 2018), sorghum (Zheng and Li, 2019), peony (Zhang et al., 2022), banana (Sánchez Moreano et al., 2021), and kale (Zeng et al., 2022). Based on structural domain homology analysis, the *TCP* gene family can be divided into two taxa, Class I (or *TCP*-P) and Class II (or *TCP*-C) (Martin-Trillo and Cubas, 2010). Class I is also known as the *PCF* subfamily, while Class II is further divided into two subfamilies, *CIN* and *CYC*/*TB1* (Lin et al., 2016). Members of the *TCP* family were found to be extensively involved in regulating plant growth and developmental processes, such as flower asymmetry (Zhao et al., 2020), branching (Braun et al., 2012), seed germination (Resentini et al., 2014), gametophyte development (Takeda, 2006), leaf development (Koyama et al., 2017), circadian rhythm (Giraud et al., 2010), and defense response (Kim et al., 2014).

The *TCP* transcription factor family is widely involved in the regulation of leaf development. For example, *AtTCP2* and *AtTCP4* play a positive role in regulating leaf senescence (Schommer et al., 2008). Loss-of-function mutants of the miR319-regulated *AtTCP3*, *AtTCP4*, *AtTCP10*, and *AtTCP24* have wrinkled leaves (Nath et al., 2003). Overexpression of miR319 resulted in decreased *TCP* levels and a series of pleiotropic phenotypes such as excessive leaf cell division, upward leaf growth, leaf size discrepancy (Rodriguez et al., 2014; Karamat et al., 2021), and edge serration (Rubio-Somoza et al., 2014; Koyama et al., 2017). In *Arabidopsis* and tomato plants, the regulation of the target gene *TCP* gene by miR319 is hindered and a reduction in leaf size occurs (Palatnik et al., 2003; Ori et al., 2007). *AtTCP5* directly promotes the transcription of *AtKNAT3* (*HOMEOBOX PROTEIN KNOTTED-1-LIKE 3*) and indirectly activates the expression of *AtSAW1* (*SAWTOOTH1*) thus playing a key role in the regulation of leaf margin development (Yu et al., 2021). *AtTCP7* affects endoreplication in *Arabidopsis* leaves and hypocotyl cells through direct regulation of the essential cell cycle gene *CYCLIN D1:1* (Zhang et al., 2019). *AtTCP13* controls leaf growth mainly by inhibiting cell expansion (Hur et al., 2019). *AtTCP14* and *AtTCP15* regulate internode length and leaf shape in plants by promoting cell proliferation (Hur et al., 2019). There is a high degree of functional redundancy among different genes in the *AtTCP* family. *AtTCP7*, *AtTCP8*, *AtTCP22*, and *AtTCP23* can interact with each other in the yeast two-hybrid assay and control leaf traits by regulating the expression of the *AtKNOX1* (*CLASS I KNOTTED-LIKE HOMEOBOX*) gene (Aguilar-Martinez and Sinha, 2013).

The regulation of TCP for plant leaf development is closely related to plant hormones. AtTCP4 can directly activate *YUCCA5* transcription and integrate the auxin response into the brassinosteroids signaling pathway to promote hypocotyl cell elongation in *Arabidopsis* (Challa et al., 2016). AtTCP4 and the chromatin remodeling factor BRM interact in plants to jointly bind the promoter of the cytokinin transcription factor *ARR16* (*ARABIDOPSIS RESPONSE REGULATORS 16*) and induce its expression to reduce leaf sensitivity to cytokinin and promote leaf growth (Efroni et al., 2013). AtTCP4 also directly regulates the expression of the jasmonic acid biosynthesis *LOX2* (*LIPOXYGENASE 2*) gene (Schommer et al., 2008). AtTCP3 activates the expression of the auxin signaling repressor *IAA3*/*SHY2* (*INDOLE-3-ACETICACID3/SHORT HYPOCOTYL 2*) (Tomotsugu Koyama and Motoaki, 2010), while the interaction of AtTCP3 with R2R3-MYBs leads to an increase in flavonoid content, which further negatively affects the response to auxin (Li and Zachgo, 2013). The regulation of CIN-TCP for leaf development is related to the expression of *NGA3* (*NGATHA 3*) because CIN-TCP can bind the promoter of *NGA3* (Ballester et al., 2015) and NGA factors are essential for auxin synthesis (Martínez-Fernández et al., 2014). *Arabidopsis* leaves overexpressing *AtNGA* have smoother leaf margins (Lee et al., 2015). *AtTCP* and *AtNGA* co-knockdown resulted in persistent expression of the distal axis gene *AtPRS* (*PRESSED FLOWER*) and the proximal axis gene *AtWOX1* at the mature leaf margin, promoting leaf margin development (Alvarez et al., 2016). AtTCP20 regulates different stages of leaf development by regulating the expression of *AtLOX2* and JA signaling (Danisman et al., 2012).

Chinese kale (*Brassica oleracea* cv. *‘XiangGu’*) belongs to the genus *Brassica* in the family *Cruciferae*. Chinese kale has leaves or flowering shoots as edible organs and contains high levels of nutrients such as ascorbic acid, minerals, and glucosinolates (Zhao et al., 2021). The diverse leaf morphology of kale is an important basis for distinguishing different variety types. ‘XiangGu’ Chinese kale (XG) is commonly known as ‘metamorphic leaves’ because of the peculiar morphology of the small leaves that grow on the veins of the true leaves (Wang et al., 2011). Previous analysis of the gene family of *BoTCP* in Chinese kale showed that *BoTCP25* (a homologous gene of *Arabidopsis AtTCP4*) was abundantly expressed in the leaves of XG. Expression analysis of different parts of the XG leaves showed that *BoTCP25* was highly expressed at the leaf margin, suggesting that it may be associated with XG leaf development (Zeng et al., 2022). To further investigate the function of BoTCP25 in XG leaf development, the overexpression vector of *BoTCP25* was constructed and *Arabidopsis thaliana* overexpression *BoTCP25* plants and XG overexpression *BoTCP25* plants were obtained in this study. The effect of the heterologous expression on plant leaf development was clarified by leaf phenotype analysis of overexpression plants, and the effect of overexpression of *BoTCP25* on leaf growth was confirmed. The use of yeast monohybrid verified that BoTCP25 could bind the promoter of *BoNGA3* (homologous gene of *AtNGA3*) *in vitro* and regulate the expression of *BoNGA3* in leaves. Finally, the reponse of the *BoTCP25* promoter to auxin and ethylene was confirmed by GUS staining, indicating that the expression of *BoTCP25* could be regulated by auxin and ethylene signal.

## Materials and methods

### Plant materials and growth conditions

The seed of the Chinese kale (*Brassica olerac*ea cv. *‘XiangGu’*) was purchased from Jieyang Nongyou Seed Co. The seeds were evenly scattered in Petri dishes (diameter = 15 cm) filled with moist perlite and placed in a light incubator (dark) at 28°C for germination, and the seedlings were transplanted in a pot containing a mixed substrate (peat: vermiculite: perlite = 3:1:1) after one week of light culture (16 h light/8 h dark). The seedlings were placed in a chamber with the following parameters: temperature 25°C, humidity 65%, and photoperiod (16 h light/8 h dark). For the genetic transformation, Chinese kale seeds were rinsed in running water for 1.5 h, disinfected with 75% alcohol for 30 min, sterilized with sodium hypochlorite for 6 min, and washed in sterile water five times (1 min each), then inoculated on 1/2 MS medium and cultured in a chamber (25°C, 16 h light/8 h dark) for 5 d to obtain the sterile seedlings.

Tobacco seeds were sown in trays and placed in a chamber at 25°C, 65% humidity, and photoperiod (16-h light/8-h dark). Three-week-old tobacco was used for subcellular localization. Seeds of wild-type (WT) and transgenic *Arabidopsis* were sterilized and planted on 1/2 MS medium, vernalized at 4°C for 2 d, and then cultured in a chamber (25°C, 16-h light/8-h dark), and seedlings were transplanted in the substrate. For the hormone treatment, different concentrations (0 μM, 100 μM, and 200 μM) of NAA (1-Naphthylacetic acid, Aladdin, N118453) and ETH (Ethephon, Merck KgaA, C0143) were applied to 25-day-old *Arabidopsis* seedlings, respectively. The samples were collected at 0h, 3h, 6h, 12h, and 24h for subsequent analysis.

### Bioinformatics analysis of the *BoTCP25* gene and its promoter

GSDS2.0 (http://gsds.cbi.pku.edu.cn/) was used to characterize the intron and exon of *BoTCP25* gene structure; NCBI online database (https://www.ncbi.nlm.nih.gov/) was used to identify and download homologous genes of *BoTCP25* in different species. DNAMAN 9.0 software was used for comparative analysis of its amino acid sequences. MEGA X software was used to construct a phylogenetic tree of BoTCP25 with homologous proteins of other species. TBtools 1.09876 software was used to extract 2,000 bp upstream of the *BoTCP25* gene start site as the promoter sequence. The type and number of cis-acting elements of the promoter were predicted online using PlantCARE Database (http://bioinformatics.psb.ugent.be/webtools/plantcare/html/).

### Cloning and vector construction of the *BoTCP25* gene and its promoter

The Chinese kale *BoTCP25* gene sequence was obtained from Ensembl Plants (http://plants.ensembl.org/) by searching for *Brassica oleracea* genome information. RNA from Chinese kale leaves was extracted and reverse transcribed into cDNA by using TaKaRa kit RNAiso Plus (Code No. 9109), PrimeScriptRTreagent Kit with gDNA Eraser (Code No. RR047A), and TB Green^®^Premix Ex TaqTMII (Code No. RR820A). Specific primers were designed using Snapgene 4.3.6 software (**Supplemental Table 1**) and cloned using a high-fidelity enzyme (10135ES60) from Yi Sheng Biotechnology (Shanghai) Co. The PCR reaction system was 50 μL, and the PCR amplification procedure was: pre-denaturation 98°C for 3 min; denaturation 98°C for 10 s, annealing 60°C for 20 s, extension 72°C for 3 s, 35 cycles; final extension 72°C for 5 min. The insert *BoTCP25* (GenBank accession number OK538880) was homologously recombined with the linearized pCAMBIA1302 vector using NcoI and SpeI nucleic acid endonucleases from NEB, and the one-step rapid cloning kit (10911ES20) from Yisheng Biotechnology (Shanghai) Co. The plasmid was then transformed into *DH5α* and positive colonies were picked for PCR. The plasmids were transformed into *Agrobacterium* after successful sequencing comparisons. The plant DNA extraction kit (18801ES50) from Yisheng Biotechnology Co., Ltd (Shanghai) was used to extract kale leaf DNA for cloning the promoter sequence of the *BoTCP25* gene (**Supplemental Table 1**). The pCAMBIA1301 vector was double cut using NEB’s SalI and NcoI nucleic acid endonucleases, and the cloning kit and sequencing were performed as above.

### Genetic transformation of BoTCP25 in Arabidopsis and Chinese kale

The pCAMBIA1302 recombinant vector was transformed into Arabidopsis Col-0 wild type by flower dip method. The T1 generation seeds were harvested and sown on 1/2 MS solid medium containing hygromycin (20 mg/L) for cultivation, and after about 20 d, the healthy growing seedlings were selected and transplanted to the monoculture harvest, which was continuously screened by hygromycin to T3 generation for subsequent experiments.

According to the pre-established genetic transformation system of Chinese kale, 5-day-old kale hypocotyls were obtained as explants and precultured for three days, and then cultured by *Agrobacterium* for two days, after which the explants were sequentially transferred to a differentiation medium containing graded hygromycin concentrations for growth. When the adventitious shoots of resistant seedlings grow to 2-3 cm, they are cut off and the adventitious roots are induced on the rooting medium, then acclimatized after rooting, and finally transferred to the nutrient substrate for normal management until flowering and seed set. The detection of positive transgenic plants was performed by referring to the method of Cao et al (Cao and Yi, 1995).

### Subcellular localization analysis of BoTCP25 protein

The pCAMBIA1302-*35S*::GFP empty vector and pCAMBIA1302-*35S*::BoTCP25-GFP were transformed into Agrobacterium tumefaciens GV3101 by freeze-thaw method and transiently transfected tobacco leaves. The fluorescence signal of the BoTCP25 protein in cells was observed using an Olympus-FV1200 laser confocal microscope (Shibuya, Japan).

### Phenotypic observations and determination of physiological indicators in transgenic plants

The phenotypes of transgenic *Arabidopsis* and kale plants were observed at 5, 25, and 45 days, and leaf-related physiological indicators were measured. RNA was also extracted from different parts of wild-type and transgenic Arabidopsis at different times, and the relative expressions of homologous genes of *BoTCP25*, *BoNGA3*, and *Bopre-miR319a* were analyzed.

Three strains of transgenic *Arabidopsis* T3 generation (L2, L5, and L6) and wild type were sown on 1/2 MS solid medium. The root phenotypes of the transgenic Arabidopsis plants were observed on day 5; the morphological phenotypes of the leaves of the transgenic *Arabidopsis* plants were observed on day 25, while the relevant physiological indicators of the leaves of 30 plants from three strains were measured, including the number of leaves, leaf length, leaf width, leaf circumference, leaf area, and plant fresh weight.

After the successful domestication and transplanting of DNA-identified F1 generation transgenic Chinese kale plants, the phenotypes of wild-type and overexpressed *BoTCP25* Chinese kale plants were observed at day 45 of the vegetative growth period. The F2 generation seeds of the above transgenic plants were obtained, and the leaf morphological phenotypes, including leaf curl rate and ectopic growth of metamorphic leaves, were observed on the transgenic F2 generation plants at day 20 and day 35, respectively. Leaf curl rate is the ratio of the number of curled leaves to the total number of leaves in the plant. Metamorphic leaves are attached at 1/2-2/3 of the main leaf veins from the petiole, and those not in that position are called ectopic growth. The plants counted were three independent replicate strains of 30 plants each in the F2 generation.

### Histochemical staining of BoTCP25-GUS and BoNGA3-GUS for tissue localization

The gene sequence of *BoNGA3* (Bo5g002790) was extracted from the *Brassica oleracea* genome (http://plants.ensembl.org/) and 2,000 bp upstream of the ATG of BoNGA3 was selected as the promoter sequence for primer design (**Supplemental Table 1**). The pCAMBIA1301 expression vector containing the GUS reporter gene was double cleaved using SalI and NcoI nucleic acid endonucleases from NEB, and the cloning ligation kit and transformation were performed as above.

GUS staining solution was prepared including sodium phosphate buffer (pH = 7.0), 0.25 M Na_2_EDTA·2H_2_O, 50 mM K_3_Fe(CN)_6_, 50 mM K_4_Fe(CN)_6_, 0.1% TritonX-100, and 100 mM X-Gluc. The transgenic plants were placed in centrifuge tubes and GUS staining solution was added to submerge the plants. The plants were incubated at 37°C for 1-24 h. As the incubation time increased, different tissue parts of the transgenic plants gradually showed blue color. The staining was observed every 0.5-1h to prevent over-staining. After the staining was completed, the material was immersed in 70% ethanol and left at 37°C for 1-3 h to remove chlorophyll, and this decolorization process was repeated until the green color faded completely. After decolorization, the samples were stored in 75% ethanol at 4°C. When observed under a microscope, the blue color on the white background is the GUS expression site.

### Yeast one-hybrid experiment verification of the interaction between *BoTCP25* and *BoNGA3*

Primers were designed based on the sequence of the predicted core region of the *BoNGA3* promoter (**Supplemental Table 1**), and the core promoter fragment was homologously recombined with the linearized pAbAi vector to construct a decoy vector. The pAbAi-p53 vector was used as a positive control and transformed into the Y1HGold yeast strain. After being identified as a positive colony, the most suitable AbA concentration was screened on SD/-ura (AbA) deficient medium, the positive single colony of the bait vector was selected to make receptor cells, and then the constructed prey vector pGADT7-*BoTCP25* was transferred into the receptor cells by co-transformation, and then incubated on SD/-Leu solid medium (with optimum AbA concentration) for 3-5 d at 30°C.

### Analysis of related gene expression by qRT-PCR

Primer Premier 5 software was used for qRT-PCR primer design for BoTCP25, BoNGA3, and Bopre-miR319a (**Supplemental Table 1**). RNAex (Code No. AG21101), Evo M-MLV Mix Kit with gDNA Clean for qPCR (Code No. AG11728), and SYBR^®^ Green Premix Pro Taq HS qPCR Kit (Code No. AG11701) were used to extract the total RNA from the samples, reverse transcription and qRT-PCR reactions, respectively. *BoActin* was used as the internal reference gene for real-time fluorescence quantitation by the CFX96 Real-Time PCR Detection System instrument. The reaction system was 20 μL: 2X SYBR^®^ Green Pro Taq HS Premix 10 μL, ddH_2_O 7.2 μL, 0.4 μL each of upstream and downstream primers, and cDNA template 2 μL. The reaction procedure was pre-denaturation at 95°C for 30 s, denaturation at 95°C for 5 s, and annealing at 60°C for 30 s with 40 cycles for amplification. Three biological replicates were performed for each sample. Relative expressions were calculated according to the 2^-ΔΔCt^ method and analyzed using Excel 2019 software, significance analysis was performed using the Duncan method in SPSS 26 software (p ≤ 0.05), and bar graphs were produced using Origin 2020 software.

## Results

### Characteristics and subcellular localization of BoTCP25

**Figure 1:**
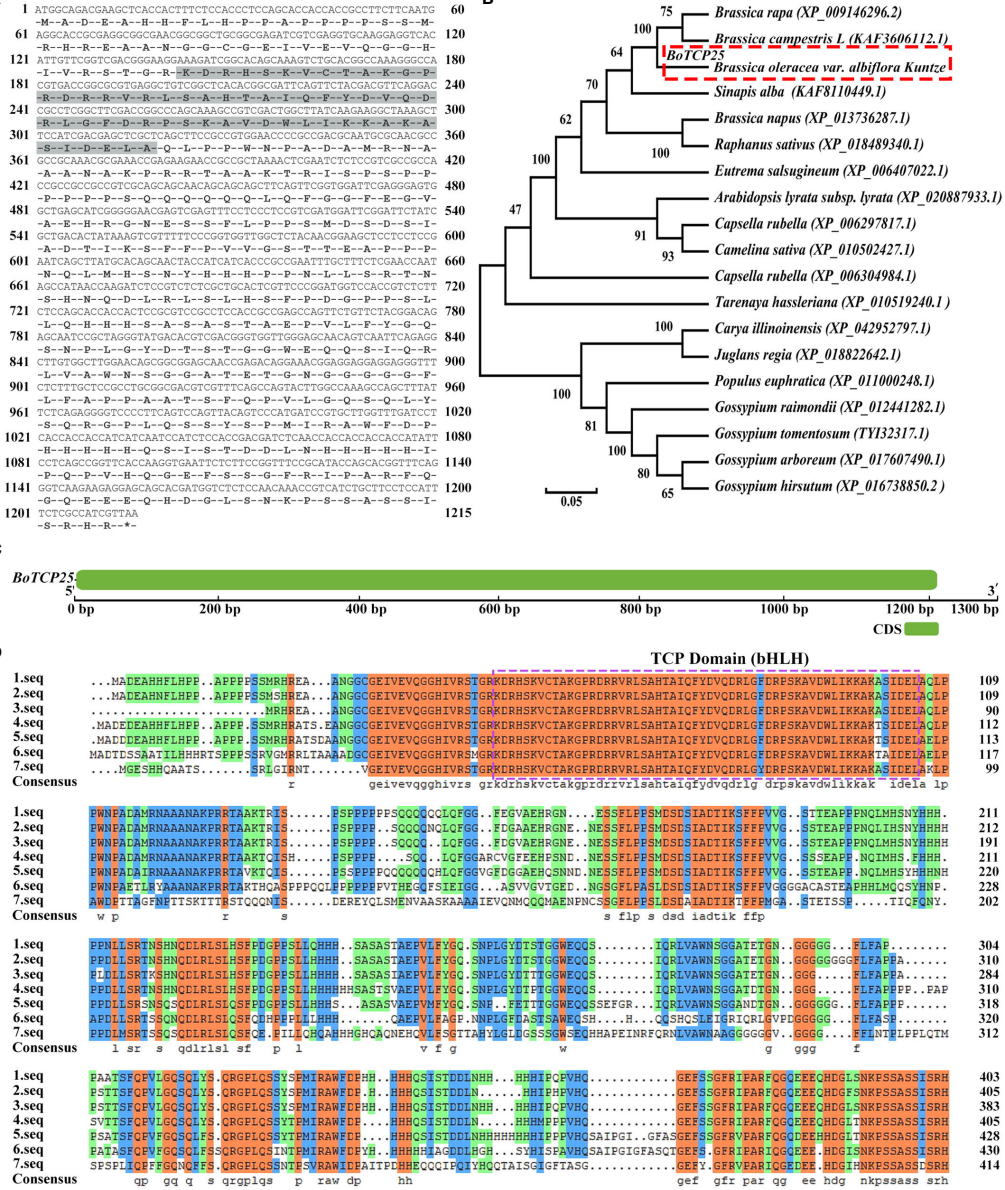
Bioinformatics analysis of BoTCP25. **(A)** Amino acid sequence encoded by BoTCP25 (the shaded area is the conserved region), **(B)** Phylogenetic tree of BoTCP25 and its homologous proteins in other species, **(C)** Gene structure of BoTCP25, **(D)** Multiple sequence alignment map of BoTCP25 and its homologous proteins in different species. 1. seq: *Brassica oleracea*; 2. seq: *Brassica rapa*; 3. seq: *Brassica campestris L*; 4. seq: *Sinapis alba*; 5. seq: *Eutrema salsugineum*; 6. seq: *Tarenaya hassleriana*; 7. seq: *Populus euphratica*.

To clarify the function of *BoTCP25* in Chinese kale, the sequence of *BoTCP25* was analyzed (**Figure 1**). The *BoTCP25* gene was successfully amplified using PCR technology at a size of 1,215 bp. The sequence of *BoTCP25* has been uploaded to the NCBI and obtained the gene registration number OK538880. Bioinformatic analysis of the *BoTCP25* gene revealed that it encodes 404 amino acid residues, with its TCP structural domain in grey (**Figure 1A**). The results of phylogenetic analysis show that *BoTCP25* has the closest affinity to the TCP25 protein in *Brassica campestris L* and *Brassica napus* and is more distantly related to the TCP25 protein in *Gossypium raimondii* (**Figure 1B**). Analysis of the structural domain of the gene revealed that it contains one exon with an intron-poor evolutionary structure with zero introns (**Figure 1C**). Multiple sequence comparisons between BoTCP25 and homologs of other species indicate the presence of a conserved TCP structural domain and presumably functional similarity between sequences of high similarity (**Figure 1D**).

To know the subcellular localization of *BoTCP25*, the pCAMBIA1302-*35S*::BoTCP25-GFP was constructed (**Supplemental Figures 1A2, 1A3**). The expression of pCAMBIA1302-*35S*::BoTCP25-GFP was observed by laser confocal microscopy using Agrobacterium infestation injected into tobacco epidermal cells (**Supplemental Figure 1B**). The results showed that tobacco epidermal cells transfected with the fusion expression vector containing the target fragment *BoTCP25* showed green fluorescence mainly in the nucleus (**Supplemental Figure 1B1**), while epidermal cells of tobacco transfected with the empty vector were covered with green fluorescence (**Supplemental Figure 1B2**).

### Analysis of the localization of *BoTCP25* in *Arabidopsis* plants

**Figure 2:**
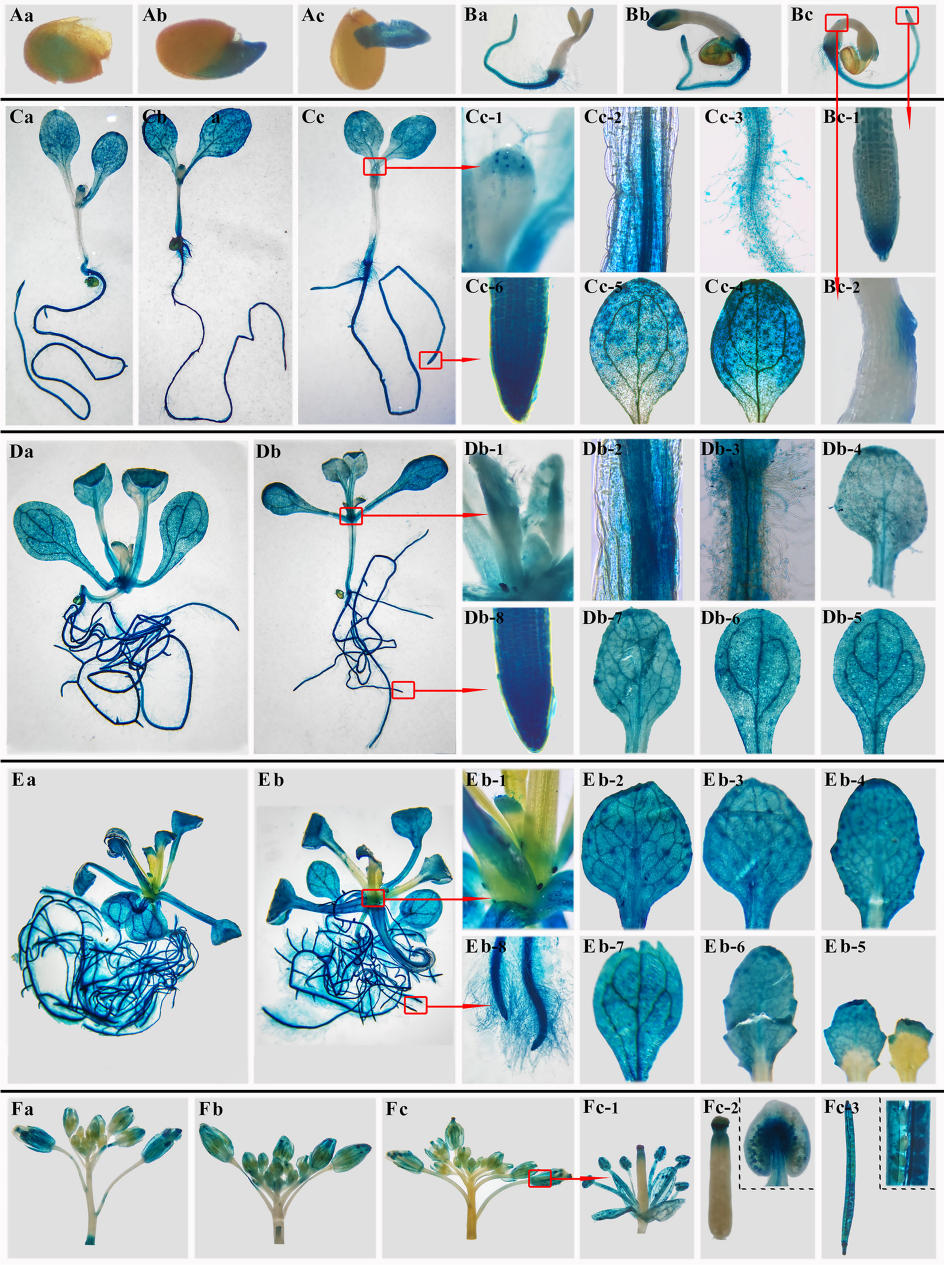
GUS staining of pCAMBIA1301-*BoTCP25pro*-GUS *Arabidopsis* plants at different stages. **(A)** Germinated seeds. **(B)** 3-day-old seedlings. **(C)** 5-day- old seedlings. **(D)** 10-day-old seedlings. **(E)** 15-day-old seedlings. **(F)** 35-day-old seedlings. Lowercase letters (a, b, c) refer to the three independent repeats of each stage. For **(D)** and **(E)**, two repeats were listed.

To detect the localization of *BoTCP25* in plants, pCAMBIA1301-*BoTCP25*-pro::GUS was constructed and transferred to *Arabidopsis thaliana* by flower dipping. The T3 generation *BoTCP25*-pro::GUS at six developmental stages was used to conduct the GUS histochemical staining (**Figure 2**). The results showed that the *GUS* gene driven by the promoter of the *BoTCP25* gene was mainly expressed in embryos in germinated seeds (**Figures 2Aa–c**). During the 3^rd^ day, *BoTCP25* was highly expressed mainly in the root hairs and root tips, with lower expression at the margins of the stem and in the cotyledons. (**Figures 2Ba–c**). During the vegetative growth stage (5, 10, and 15 DAS), *BoTCP25* was expressed at high levels in all tissue such as leaf margin and leaf flesh, stem tip, stem vascular tissue, root and root hairs, and apical region (**Figures 2C–E**). When it comes to reproductive growth (30 DAS), *BoTCP25* shows high expression in buds, pistils, and anthers, as well as in young seed pods (**Figures 2Fa–c**). In conclusion, the *BoTCP25* promoter-driven *GUS* gene was expressed in different tissues, both during vegetative and reproductive growth, indicating that its expression was widespread and not tissue-specific.

### Heterologous expression of *BoTCP25* promotes the growth of a plant

**Figure 3:**
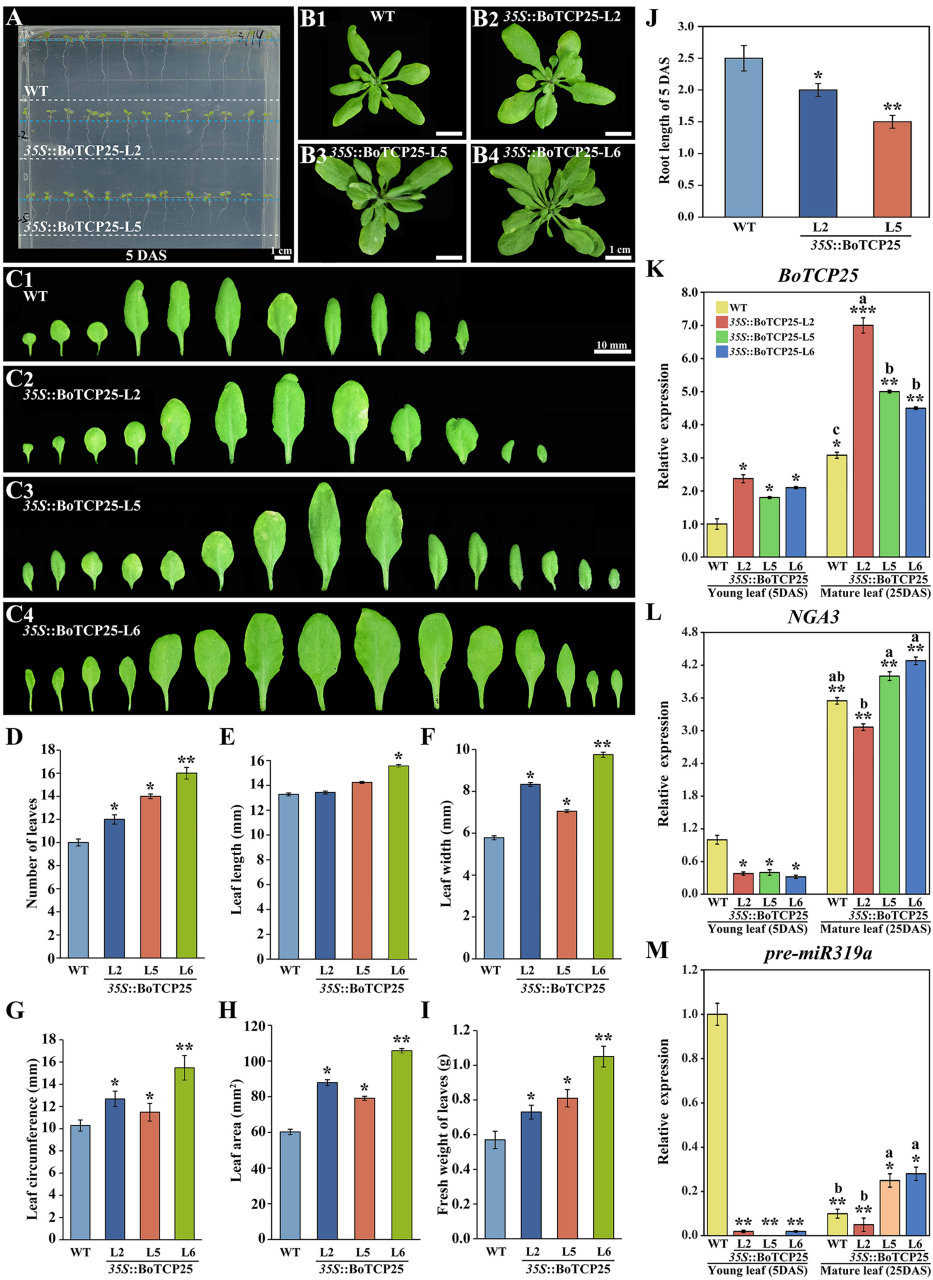
Phenotypic analysis of ectopic expressed BoTCP25 *Arabidopsis* plants and relative expression analysis of related genes. Phenotypic analysis of **(A)** 5-day-old and **(B)** 25-day-old pCAMBIA1302-*35S*::BoTCP25-GFP transgenic plants and the corresponding wild type. **(C)** Comparison of dissected leaves of three *BoTCP25-GFP* transgenic lines and the corresponding wild type. Statistical analysis of the number of leaves **(D)**, leaf length **(E)**, leaf width **(F)**, leaf circumference **(G)**, leaf area **(H)**, and fresh weight **(I)** of *BoTCP25-GFP* transgenic plants and the wildtype. **(J)** Root length of 5-day-old *BoTCP25-GFP* transgenic plants and the wild type. Relative expression of *BoTCP25* **(K)**, *NGA3* **(L)**, and pre-miR319a **(M)** in different parts of *BoTCP25-GFP* transgenic plants. The significant difference in leaves was marked with an asterisk (*). A single asterisk (*) means p<0.05, double asterisks (**) mean p<0.01, and three asterisks (***) mean p<0.001. Different lowercase letters represent a significant difference in mature leaves (p<0.05). The bar in A and B is 1 cm, and C is 10 mm. DAS, Days After Sowing; WT, wild type. L2, L5, and L6 are three *BoTCP25-GFP* transgenic *Arabidopsis* lines.

Preliminary bioinformatics and qRT-PCR experiments have shown that transcription factor *BoTCP25* is closely related to leaf development (Zeng et al., 2022). To verify the function of *BoTCP25* in leaf development, the pCAMBIA1302-*35S*: BoTCP25-GFP recombinant vector was transferred to *Arabidopsis thaliana*, and the T3 homozygous plant was used for subsequent phenotypic analysis (**Figure 3**). Five-day-old *Arabidopsis* seedlings expressing *BoTCP25*-GFP exhibited shorter roots compared with the wild type (**Figure 3A**). During the vegetative growth period, the 25-day *BoTCP25*-GFP transgenic strains have more leaves and larger foliage (**Figures 3B, C**). Statistical analysis verified the role of *BoTCP25* in promoting the growth of the leaf (**Figures 3D–I**). The leaf numbers, leaf width, leaf area, and fresh weight were all increased in the transgenic plants expressing *BoTCP25*-GFP (**Figures 3D–I**). The root length was decreased in the *BoTCP25-GFP* transgenic *Arabidopsis* compared with that in the wildtype (**Figure 3J**). The expression of *BoTCP25* was higher in mature leaves than in young leaves in both wildtype and transgenic plants (**Figure 3K**). In all three transgenic lines, the expression of *BoTCP25* increased in young leaves and mature leaves to varying degrees (**Figure 3K**), indicating that the phenotypic changes in *BoTCP25*-GFP transgenic lines were caused by the upregulation of *BoTCP25*.

To further clarify the regulation mechanism of *BoTCP25* in leaf development, the downstream genes of *BoTCP25* were screened based on the published data, and *NGA3* was selected for its key role in leaf development (Ballester et al., 2015). The expression of *NGA3* was downregulated in five-day-old seedling leaves of *BoTCP25*-GFP (**Figure 3L**). For the mature leaves, the expression of *NGA3* did not change significantly relative to the wildtype. Besides, the expression of *TCP4* was regulated by miR319 in *Arabidopsis* (Schommer et al., 2014), thus the expression of miR319’s precursor was analyzed in the transgenic lines (**Figure 3M**). The abundance of miR319 was decreased in mature leaves compared with the young leaves in wildtype and the overexpression of *BoTCP25* reduced the transcripts of miR319 in young leaves. In mature leaves, the expression of miR319 was increased in L5 and L6 transgenic lines while did not change in the L2 line compared with the wildtype.

### Overexpression of *BoTCP25* cause leaf curl and ectopic growth of metamorphic leaves in Chinese kale

**Figure 4:**
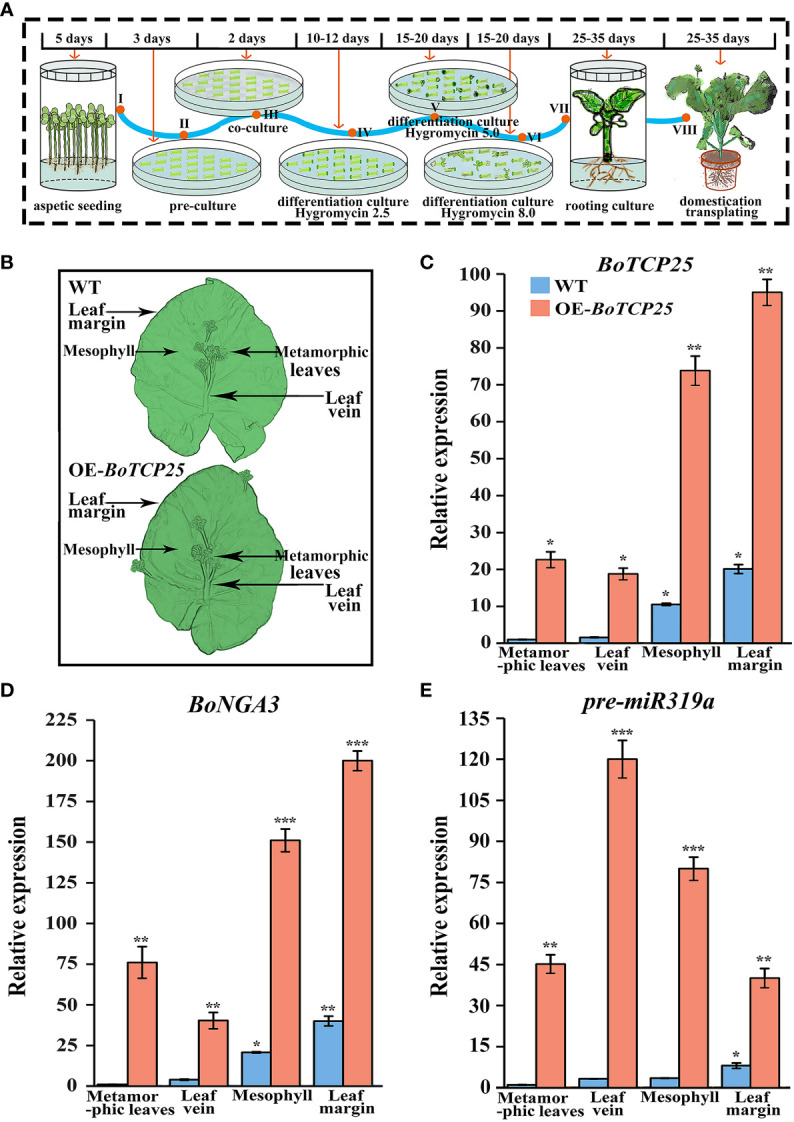
Identification of transgenic plants and gene expression of overexpressed *BoTCP25-GFP* XG plants. **(A)** Schematic diagram of genetic transformation of BoTCP25 to XG Chinese kale. **(B)** Diagram of sampling site of Chinese kale plant leaves. The expression of *BoTCP25* **(C)**, *BoNGA3* **(D)**, and pre-miR319a **(E)** in different parts of the XG leaf. Bars are equal to 100 mm. *p<0.05, **p<0.01, and ***p<0.001.

To illustrate the regulatory role of *BoTCP25* on leaves of Chinese kale, we have established a genetic transformation system and transferred pCAMBIA1302-*35S*::BoTCP25-GFP to Chinese kale (**Figure 4**). The schematic procedure for establishing the genetic transformation system were listed in **Figure 4A**. The whole process of genetic transformation lasts for 100-132 days. After domestication and transplantation, the DNA of the leaf was extracted and the expression of *BoTCP25* was tested to obtain the genetically modified plants. The leaves of XG Chinese kale were divided into four parts (**Figure 4B**), including metamorphic leaves, main leaf vein, mesophyll, and leaf margin for sampling, and then the expression levels of related genes were analyzed (**Figures 4C–E**). The results showed that the expression of *BoTCP25* was elevated in all four parts of the leaves of the transgenic plants, with the most elevated in the leaf margins (**Figure 4C**). The expression of *BoNGA3* followed a similar pattern to that of TCP, with the highest expression at the leaf margins (**Figure 4D**). Different from the expression pattern of *BoTCP25*, the transcripts of pre-miR319a were highest in the leaf veins, and lowest in the leaf margins (**Figure 4E**).

**Figure 5:**
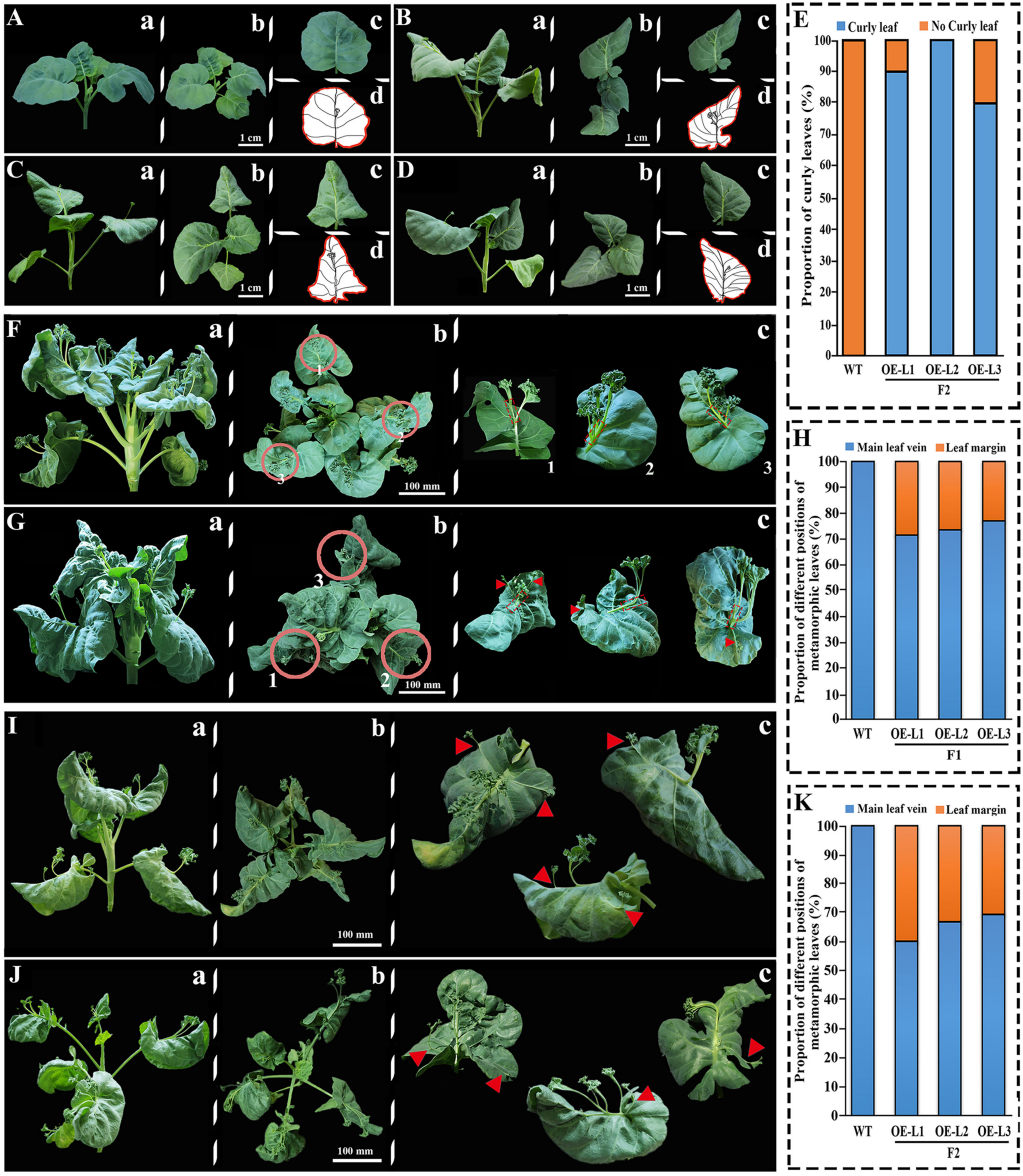
Phenotypic analysis of overexpressed *BoTCP25-GFP* XG plants. The phenotype of leaves of 20-day-old wild-type **(A)** and three lines of F2 transgenic *BoTCP25-GFP* XG plants **(B-D)**. **(E)** The proportion of curly leaves in the wild type and BoTCP25-GFP overexpressed lines. The metamorphic leaves in the wild-type **(F)** and F1 *BoTCP25-GFP* overexpressed Chinese kale plant generation **(G)**. **(H)** The proportion of metamorphic leaves on the main leaf vein and margin in the wild-type and *BoTCP25-GFP* F1 generation transgenic plant. **(I-J)** Typical metamorphic leaves on the *BoTCP25-GFP* transgenic plants. **(K)** The proportion of metamorphic leaves on the main leaf vein and margin in the wild-type and *BoTCP25-GFP* F2 generation transgenic plant. Lowercase letters a, b, and c refer to the frontal, top, and single-leaf views of each stage. The lowercase letter d is the diagram of a single leaf in **(C)** The red triangle is the site of the metamorphic leaves. The bars in **(A–D)** indicate 1 cm, and in **(F, G, I)**, and **(J)** 10 cm. DAS, Days After Sowing; DPI, Day Post Inoculation.

Statistical analysis of F1 and F2 phenotypes of transgenic XG Chinese kale (**Figure 5**). We found that at 20 DAS, the leaves of transgenic plants curled downward (**Figures 5B–D**) relative to the wild-type plants (**Figure 5A**), and the leaves of the three lines showed varying degrees of curl (**Figure 5E**), with L1 strain at 90%, L2 strain at 100%, and L3 strain at 80%.

The main difference in metamorphic leaves between the control and transgenic XG Chinese kale was represented in the diagram (**Figure 5**). The wild type of XG Chinese kale possesses metamorphic leaves at 1/2-2/3 of the main leaf vein as its character (**Figure 5F**). In contrast, the transgenic XG Chinese kale expressing *35S*::BoTCP25-GFP exhibited a reduced number of deformed leaves at the main leaf vein while the appearance of metamorphic leaves was noticed near the leaf margins (**Figures 5G, I, J**). Further statistics were conducted on the location of the ectopic growth of the metamorphic leaves, which found that 20%-30% of the metamorphic leaves in the 45 DPI (Day Post Inoculation) (**Figure 5G**) had ectopic leaf margin growth (**Figure 5H**), In the 35 DAS (Day After Sowing) of metamorphic leaves of F2 generation transgenic plants (**Figures 5I, J**), it increased to reach 30%-40% (**Figure 5K**).

### The interaction between BoTCP25 and *BoNGA3*

**Figure 6:**
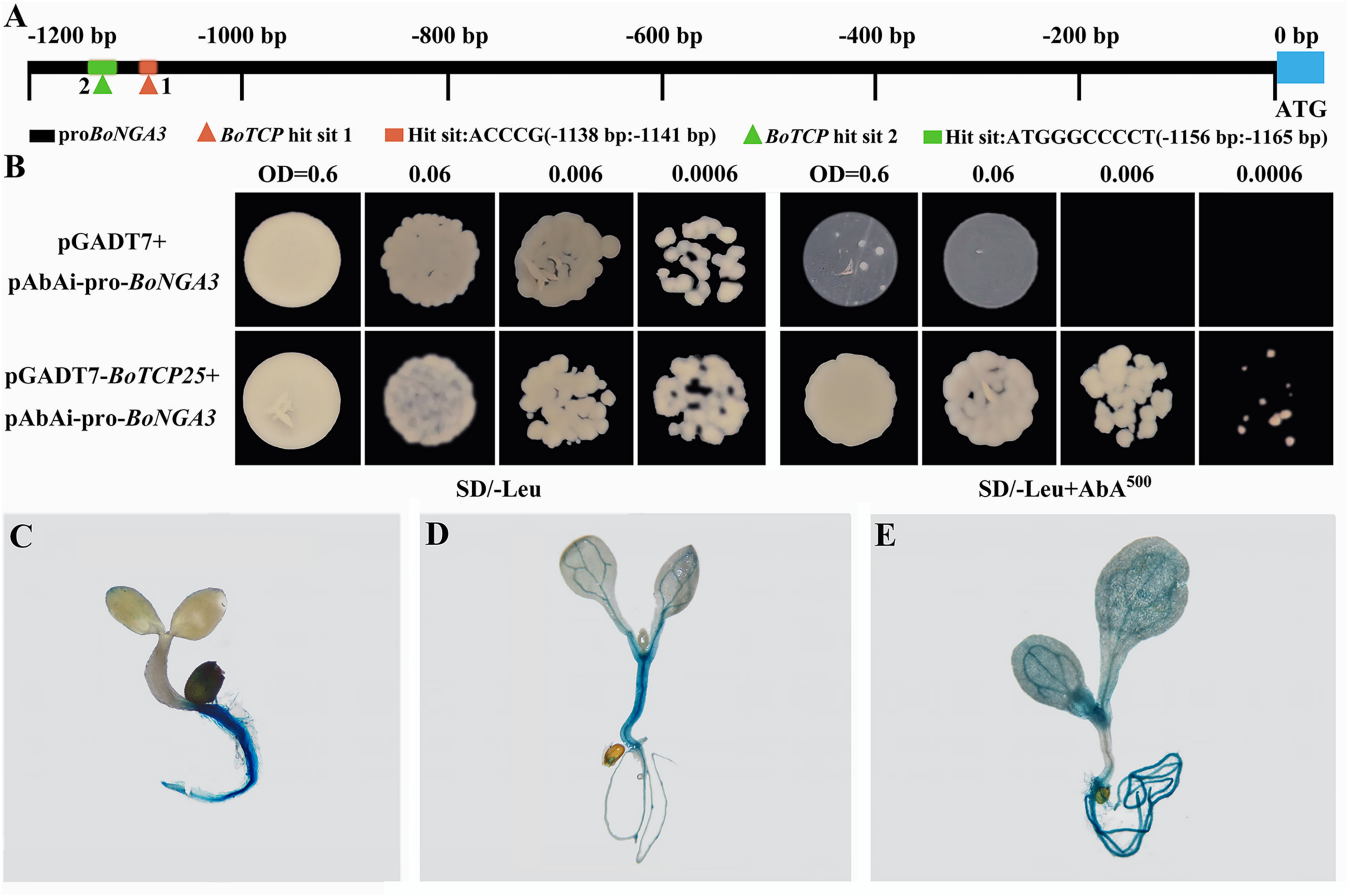
Yeast one hybrid and GUS staining of *BoNGA3 promoter*-GUS transgenic *Arabidopsis* plants. **(A)** Schematic diagram of potential interaction sites between BoTCP25 and BoNGA3’s promoter. **(B)** Verification of the binding of BoTCP25 to the promoter of BoNGA3 by Yeast one hybrid method. GUS staining of *BoNGA3 promoter*-GUS transgenic *Arabidopsis* plants at 3 DAS **(C)**, 5 DAS **(D)**, and 10 DAS **(E)**. DAS, Days After Sowing.

The consistent expression pattern of *BoTCP25* and *BoNGA3* in transgenic XG Chinese kale was noticed, however, it remains unknown whether they function together in the induction of deformed leaf at the leaf margin. Yeast-one-hybrid experiment was performed to test the interaction between BoTCP25 and BoNGA3 (**Figure 6**). The promoter of *BoNGA3* was cloned and two binding sites of BoTCP2 were predicted using PlantPAN 3.0 (**Figure 6A**). Based on the optimal AbA (500 ng/ml) concentration screened in SD/-ura medium, the prey vector pGADT7-BoTCP25 and the negative control pGADT7 plasmid were co-transformed into the pAbAi-*proBoNGA3* strain in SD/-Leu medium containing 500 ng/mL AbA, respectively. A single colony was then cultured at gradient dilution and the negative control barely grew with increasing dilution, while the positive co-transformed colonies (pGADT7-BoTCP25 and pAbAi-*proBoNGA3*) were able to grow normally (**Figure 6B**). The 2000-bp BoNGA3 promoter sequence was constructed into the pCAMBIA1301 vector containing the GUS reporter gene and the obtained T3 generation plants were stained for GUS staining (**Figures 6C–E**). The results showed that by day 3, *BoNGA3* had high expression in root hairs and root tips and no expression in stems and cotyledons (**Figure 6C**). By days 5 (**Figure 6D**) and 10 (**Figure 6E**), *BoNGA3* was abundantly expressed in root hairs, root tips, and leaf veins of the plants, but not in the leaf flesh.

### *BoTCP25* responds to exogenous hormone treatment

**Figure 7:**
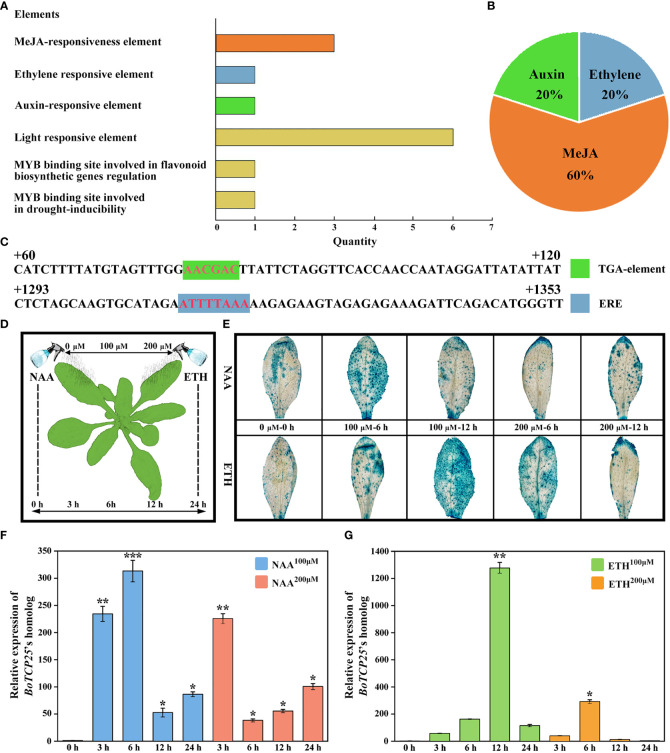
Analysis of cis-acting elements of BoTCP25’s promoter and their response to exogenous hormone treatments. **(A)** Prediction of cis-acting elements of *BoTCP25*’s promoter. **(B)** Percentage of cis-acting elements of *BoTCP25*’s promoter in response to different plant hormones. **(C)** Analysis of potential binding sites of auxin and ethylene on the *BoTCP25* promoter. **(D)** Diagram of exogenous hormone treatment on pCAMBIA1301-*BoTCP25 pro*-GUS *Arabidopsis* plants. **(E)** Response of *BoTCP25pro*-GUS to auxin and ethylene treatments. Relative expression of *BoTCP25*’s homolog in *Arabidopsis* under different concentrations of auxin **(F)** and ethylene **(G)** at different time points. *p<0.05, **p<0.01, and ***p<0.001.

To elucidate the regulatory mechanism of *BoTCP25*, we analyzed the cis-acting elements on the *BoTCP25* promoter sequence and their response to external signals (**Figure 7**). The results showed that there were six light response elements, three methyl jasmonate response elements, two *MYB* binding site elements, one auxin response element, and one ethylene response element on the *BoTCP25* promoter sequence (**Figures 7A–C**). To verify the response of *BoTCP25* to hormones, the leaves of pro*BoTCP25*-*GUS* plants were sprayed with 0 μM, 100 μM, and 200 μM of NAA and ETH, respectively, and sampled at 0 h, 3 h, 6 h, 12 h, and 24 h to analyze the expression of the *BoTCP25* promoter in response to hormonal changes (**Figures 7D–I**). On the one hand, *GUS* staining of leaves after 0 h, 6 h, and 12 h of treatment with different concentrations of NAA and ETH showed that blue highlights covered almost the entire leaf surface at 6 h of 100 μM NAA treatment and 12 h of 100 μM ETH treatment, indicating that the leaves responded most strongly to the hormone under these conditions (**Figure 7E**). On the other hand, the expression of *BoTCP25* in leaves at different time points in response to hormone treatment was analyzed by qRT-PCR, and the *BoTCP25*’s promoter expression was consistent with the results of *GUS* staining. The expression of *BoTCP25* was highest after 6 h treatment with 100 μM NAA (**Figure 7F**) and peaked after 12 h treatment with 200 μM ETH (**Figure 7G**).

## Discussion

Metamorphic leaves are used in many vegetables as edible organs or as a characteristic of the species. *Brassica oleracea* is a large genus containing plants with different morphologically diverse varieties and Chinese kale is one of them. There are various types of Chinese kale, among which XG has metamorphic leaves attached to the true leaves, and this metamorphic leaf becomes its characteristic. The regulation mechanism of leaf development is a complex regulatory process involving multiple genes and signaling pathways (Ichihashi and Tsukaya, 2015). However, it is unknown whether the regulatory mechanism of metamorphic leaves is the same as that of normal leaves. The *TCP* family can regulate plant organ morphology by affecting cell proliferation and differentiation (Aguilar-Martinez and Sinha, 2013) and plays a role in several aspects of plant leaf growth and development, including regulation of shoot apical meristem development (Tomotsugu Koyama and Motoaki, 2010) and modulation of changes in leaf morphology and size (Ori et al., 2007). As a homolog of TCP4, BoTCP25 participates in leaf development and is expressed differently in Chinese kale leaves (Zeng et al., 2022).

In this study, the function of the transcription factor *BoTCP25* and its effect on metamorphic leaves were analyzed using *Arabidopsis* and Chinese kale with metamorphic leaves. Chinese Kale and *Arabidopsis* belong to the same cruciferous family and may share certain similarities in leaf growth and development. However, BoTCP25 exhibits different functions in the two *Brassica* species. Overexpression of *BoTCP25* induced the ecotopic growth of metamorphic leaves in XG Chinese kale but not in *Arabidopsis*, indicating that BoTCP25 participates in changing the localization but not directly in the formation of metamorphic leaves. Besides, the heterologous transformation of *BoTCP25* did not result in metamorphic leaves in *Arabidopsis*, but only increased leaf area and leaf number, indicating that the regulation of metamorphic leaves by *BoTCP25* is dependent on the expression of the underlying gene in XG Chinese kale itself. The fact that metamorphic leaves do not form in *BoTCP25-GFP Arabidopsis* may be due to the absence of relevant regulatory elements or the presence of repressors in *Arabidopsis*. One example is the differential expression of *BoNGA3* in the *BoTCP25* overexpressed Chinese kale and *Arabidopsis*. NGA3 is a member of the B3-domain transcription factor and functions in the development of the leaf (Ballester et al., 2015). In the current study, BoTCP25 can bind to the promoter of *BoNGA3* and change its expression in the overexpression lines. In *BoTCP25* overexpressed Chinese kale, the expression of *NGA3* increases significantly while it decreases in young leaves and does not change in mature leaves of *Arabidopsis* overexpressing *BoTCP25* relative to the wildtype. The divergent expression levels of *NGA3* in *BoTCP25* transgenic Chinese kale and *Arabidopsis* suggest that *BoTCP25* may function differently in different species which in turn results in a varied phenotype.

Another gene differentially expressed in *BoTCP25* transgenic Chinese kale and *Arabidopsis* is miR319, a regulator of *TCP4*, which negatively regulates the transcription of *TCP4* and is involved in the regulation of plant leaf development (Schommer et al., 2014). In *Arabidopsis* WT, the expression of miR319a is dynamic in leaves at different stages, high in young leaves and low in mature leaves. Meantime, the expression of *TCP4* is contrary to that of miR319a, confirming the regulation of miR319a to TCP4 in leaf development. Interestingly, in *BoTCP25* overexpressed *Arabidopsis*, the transcripts of miR319 were significantly reduced in the young leaves and the miR319a was still kept at low levels in mature leaves. The response of miR319a to the overexpression of *BoTCP25* differed in Chinese kale from Arabidopsis. In *BoTCP25* transgenic Chinese kale, the abundance of miR319 increased significantly, indicating the different mechanisms triggered by overexpressing *BoTCP25* in Chinese kale and *Arabidopsis*. Furthermore, in Chinese kale overexpressing *BoTCP25*, the expression of miR319 was highest in the leaf veins, followed by mesophyll and leaf margins, whereas the transcripts of *BoTCP25* were most abundant in leaf margins, then mesophyll and leaf veins. The negatively related expression pattern of miR319 and *BoTCP25* in different parts of Chinese kale leaves suggests that the distribution of *BoTCP25* in leaves is possibly due to a gradient in the expression of miR319 in leaves. In addition, the expression trends of miR319 and *BoTCP25* in the control of Chinese kale remain consistent, which may be attributed to the fact that miR319 can target more than one CIN-TCP. However, Under the 35S promoter, *BoTCP25* still showed differential expression in transgenic *Arabidopsis* leaves, i.e., low expression in young leaves and high expression in mature leaves, and this trend was consistent with the *Arabidopsis* wild type, indicating that heterologously expressed *BoTCP25* activated the regulatory network in *Arabidopsis* and functioned in the leaf development. It is noteworthy that the factors regulating *BoTCP25* expression are not only miR319, as the relatively low expression level of miR319 in transgenic plants indicates the existence of other factors regulating BoTCP25 expression.

*BoTCP25* can rapidly respond to changes in auxin, and in the current study, heterologous overexpression of *BoTCP25* caused an increase in leaf number and an enlarged leaf area in *Arabidopsis*, yet the mechanism of the role of auxin in *BoTCP25* regulation of leaf development remains unknown. It has been shown that *TCP4* (a homolog of *BoTCP25*) is involved in the regulation of plant leaf development by inhibiting cell division and promoting cell differentiation (Challa et al., 2019; Seki et al., 2020). In lettuce (*Lactuca sativa*), reduced expression levels of *LsTCP4* cause abnormal leaf cell division and the formation of serrated or curled leaf margin phenotypes (Seki et al., 2020). TCP4 can both activate the expression of *YUCCA5*, which further causes changes in auxin-responsive genes to alter leaf size (Challa et al., 2016), and TCP4 can also directly bind to the auxin-responsive factor *HAT2* (*HOMEODOMAIN ARABIDOPSIS THALINAN 2*) to regulate leaf size (Challa et al., 2019). Furthermore, the use of dexamethasone (DEX) to control the expression of *35S*::mTCP4 (miR319 resistant TCP4) indicates that as little as 3 h overexpression of *mTCP4* is sufficient to make the plant leaf area smaller (Challa et al., 2019). This is different from the increased leaf area caused by ectopic overexpression of *BoTCP25* in this study. Notably, *35S*::mTCP4, which is linked to the DEX, was modified not to be targeted by 319; if the sites in TCP4 regulated by miR319 were normal, *35S*::TCP4 was not able to cause changes in leaf area (Challa et al., 2019), indicating that TCP4 is dependent on the miR319 regulation in the function of leaf size control. In the present study, the increase in leaf area of *Arabidopsis* caused by *BoTCP25* under *35S* may also be due to a functional deficiency of miR319 caused by feedback inhibition. As to whether the increase in leaf area in *BoTCP25* transgenic plants is a direct result of *BoTCP25* or an indirect effect, further experiments are needed to verify.

**Figure 8:**
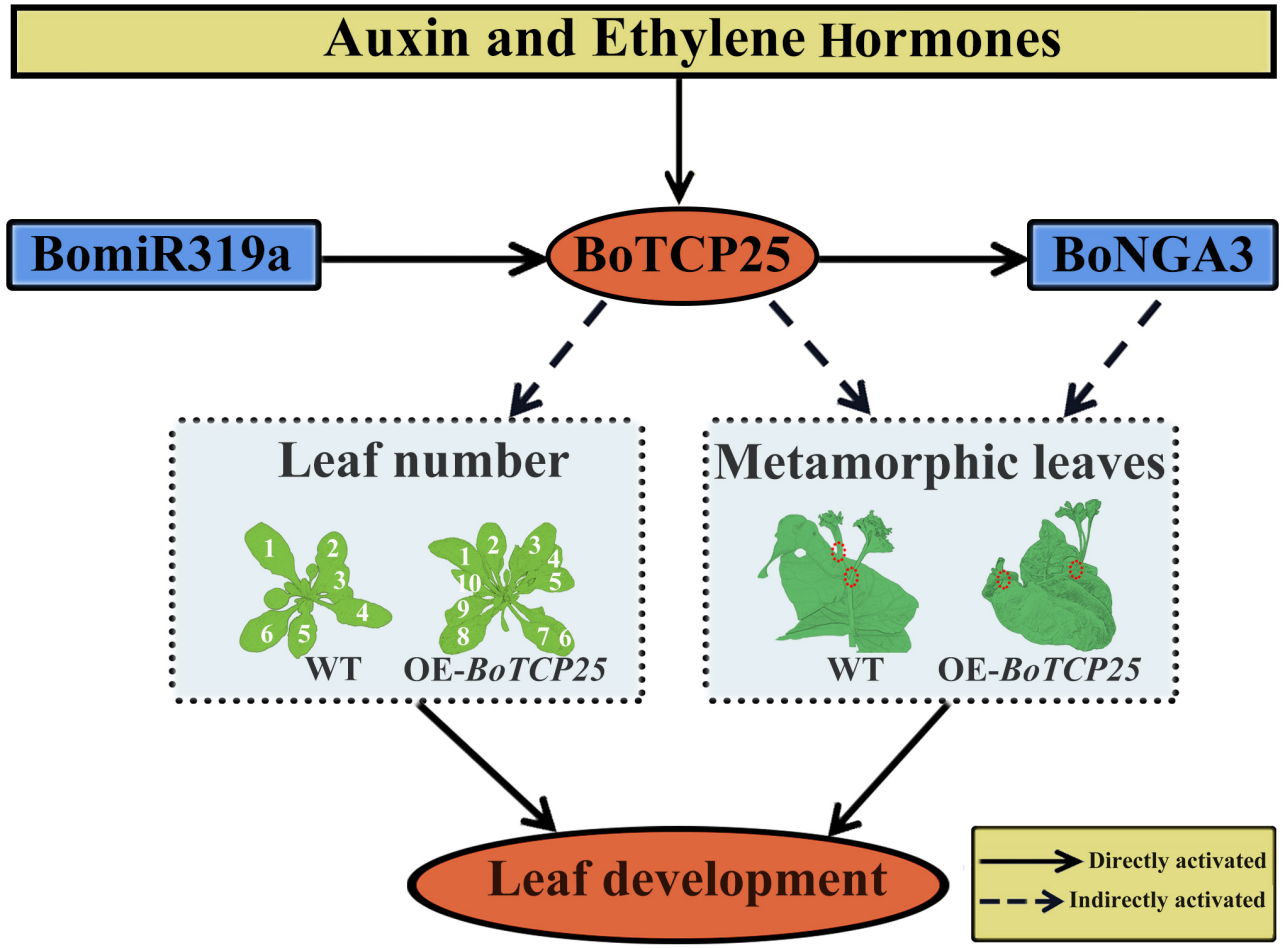
Diagram of potential regulatory mechanisms of BoTCP25 and its role in leaf development. The function of BoTCP25 in leaf development differs in *Arabidopsis* and Chinese kale. In *Arabidopsis* (left-box), BoTCP25 is related to the leaf number and leaf area, while in Chinese kale (right-box), BoTCP25 participates in changing the localization of metamorphic leaves. The expression of *BoTCP25* was regulated by miR319 and responded to the plant hormone auxin and ethylene. Besides, BoTCP25 can also bind to the promoter of *BoNGA3* to regulate the expression of *BoNGA3*. The module miR319-BoTCP25-BoNGA3 may function together in the regulation of leaf development. The number means leaf numbers in different plants. The red circle means the position of metamorphic leaves. The straight arrows are for direct action and the dashed arrows are for indirect regulation.

In conclusion, overexpression of *BoTCP25* increased the number of *Arabidopsis* leaves and caused ectopic growth of metamorphic leaves in Chinese kale (**Figure 8**). The expression of *BoTCP25* can be induced by auxin and ethylene hormone signals and is under the regulation of miR319. The expression of *BoNGA3* can be directly regulated by BoTCP25 and BoTCP25-BoNGA3 may function together in the regulation of leaf development.

## Data availability statement

The datasets presented in this study can be found in online repositories. The names of the repository/repositories and accession number(s) can be found in the article/**Supplementary Material**.

## Author contributions

RG conceived the study and revised the manuscript. JZ analyzed the data and wrote the manuscript. MY, JD, and DZ prepared materials and conducted the experiments. ZL, GW-P, and XX revised the manuscript. All authors contributed to the article and approved the submitted version.
