# Supplementary material for: The function of BoTCP25 in the regulation of leaf development of Chinese kale
Source: Front Plant Sci. 2023 Apr 18;14:1127197. doi: 10.3389/fpls.2023.1127197 (PMC10151756; doi:10.3389/fpls.2023.1127197)

**Supplemental Figure 1.** BoTCP25 gene cloning and vector construction

(A1) PCR amplification of *BoTCP25* gene, M: 2000 DNA Marker, 1-2: amplified fragments of *BoTCP25*.

(A2) pCAMBIA1302-*35S*::BoTCP25-GFP bacterial PCR, M: 2000 DNA Marker, 1-10: bacterial PCR amplification fragments.

(A3) pCAMBIA1302 recombinant vector structure.

(B1) Subcellular localization of pCAMBIA1302-*35S*::BoTCP25-GFP fusion expression vector.

(B2) pCAMBIA1302-GFP as negative control, fluorescence on the left, bright field in the middle, merged image on the right (scale bar length = 60 μm)


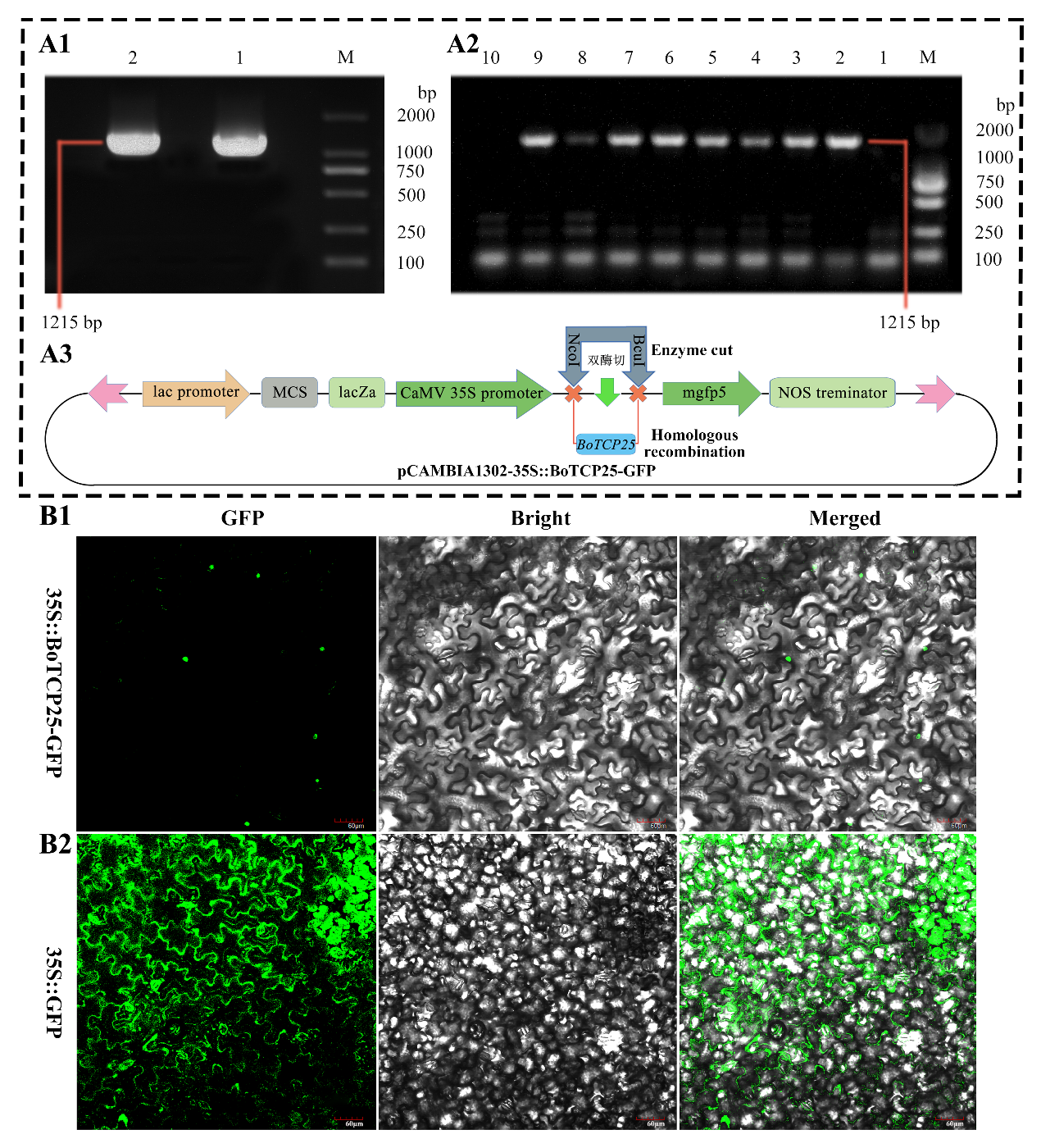

Supplement: Supplementary file 1 [file DataSheet_1.zip › Supplemental Figure 1.docx]
