# Supplementary material for: The function of BoTCP25 in the regulation of leaf development of Chinese kale
Source: Front Plant Sci. 2023 Apr 18;14:1127197. doi: 10.3389/fpls.2023.1127197 (PMC10151756; doi:10.3389/fpls.2023.1127197)
Supplement: Supplementary file 1 [file DataSheet_1.zip › Supplemental Table 1.docx]

Supplemental Table 1 Primers that were used in this study.

| Use | Primer names | Sequences（5′→3′） |
| --- | --- | --- |
| RT-qPCR | *BoActin-*F | CTGTGACAATGGTACCGGAATG |
|  | *BoActin-*R | ACAGCCCTGGGAGCATCA |
|  | *BoTCP25-*F | GTCCCCTTCAGTCCAGTTACAGT |
|  | *BoTCP25-*R | GACCATCGTGCTGCTCCTCT |
|  | *BoNGA3-*F | AGTTTGTAGGGTATGGTTATGGG |
|  | *BoNGA3-*R | GTAGAAGGAGGCGGAGGAAG |
|  | *BomiR319a-*F | TCCATTCAGAGGCTGTGATATG |
|  | *BomiR319a-*R | GCTCCCTTCAGTCCAACCAA |
| Gene cloning | *BoTCP25 -*IF | GGACTCTTGACCATGGATGGCAGACGAAGCTCACC |
|  | *BoTCP25 -*IR | CTTCTCCTTTACTAGTACGATGGCGAGAAATGGAGG |
| Bacterial PCR | *35S*-F | GTGGATTGATGTGATATCTCC |
|  | GFP-R | CTGACAGAAAATTTGTGCCC |
| Promoter cloning | *BoTCP25pro -*IF | ATCCTCTAGAGTCGACGAATTAGGGCCCCCAAGGAATTAAG |
|  | *BoTCP25pro-* IR | CTCAGATCTACCATGGTGGTTGAGCATATTGAGGAAGCTCG |
|  | *BoNGA3pro-* IF | ATCCTCTAGAGTCGACCAAGATAATGCTAAACAAAGTTCGAAAAGAAACCT |
|  | *BoNGA3pro-* IR | CTCAGATCTACCATGGGAGGTATTATATGATTTTCCTTGGAGAAATCTTGAT |
| Bacterial PCR | *M13-*R | CAGGAAACAGCTATGACC |
|  | *GUS-*R | CTATCATCATCATAGACAC |
| Yeast-one-hybrid | pAbAi*-BoNGA3pro -*IF | AAAAGCTTGAATTCGAGCTCATGGAATTTCAAACAATAACACTTCGTACGT |
|  | pAbAi-*BoNGA3*pro -IR | ATGCCTCGAGGTCGACAGAGAGAGAGGTTGAAATTGAAGTTGTCT |
|  | pGADT7*-BoTCP25 -*IF | GGAGGCCAGTGAATTCATGGCAGACGAAGCTCACC |
|  | pGADT7*-BoTCP25 -*IR | CGAGCTCGATGGATCCACGATGGCGAGAAATGGAGGA |
| DNA identification | *Hygr-*F | CGATTGCGTCGCATCGACC |
|  | *Hygr-*R | \| TTCTACAACCGGTCGCGGAG \| \| --- \| |
